# The neglect of treatment-construct validity in psychotherapy research: a systematic review of comparative RCTs of psychotherapy for Borderline Personality Disorder

**DOI:** 10.1186/s40359-016-0151-2

**Published:** 2016-08-24

**Authors:** Lars-Gunnar Lundh, Terese Petersson, Martin Wolgast

**Affiliations:** Department of Psychology, Lund University, Box 213, 221 00 Lund, Sweden

**Keywords:** Psychotherapy, Randomized controlled trials, Internal validity, Construct validity, Treatment package, Treatment model, Borderline personality disorder, Treatment contrasts, Treatment integrity, Alternative explanations

## Abstract

**Background:**

Randomized controlled trials (RCTs) are considered the best methodology for studying the efficacy of psychotherapy. Optimally an RCT design makes it possible to conclude that if one treatment has a better outcome than another, this is due to the treatment package (TP) as it was implemented in this particular context*,* rather than other factors beyond the treatment (= high internal validity). Strong internal validity does not, however, provide evidence for the treatment model (TM) that provides the theoretical basis of the TP, because the TP that is tested may differ from the comparison condition in a number of other ways that suggest alternative explanations for the effects. These alternative treatment contrasts represent threats to construct validity of the conclusions. Maximal construct validity requires (1) that the treatments are clearly contrasted on the experimental factors (treatment integrity), and (2) that alternative treatment contrasts can be eliminated. The analysis of alternative explanations is a neglected topic in psychotherapy research. To approach this problem, a methodology for the analysis of treatment contrasts is suggested and tested.

**Methods:**

Two indexes were defined: (1) a Treatment Integrity Index (TII) and (2) an Alternative Treatment Contrast Index (ATCI). This methodological approach was applied to eight comparative RCTs of treatments for Borderline Personality Disorder (BPD), which were coded for a set of treatment contrasts independently by three coders.

**Results:**

The analysis of the RCTs of treatments for BPD showed that construct validity differed widely between the different studies but was generally low (low TII and ATCI), and that it is therefore difficult to draw causal conclusions from this research. The publication policies of scientific journals in this area seldom require the systematic data relevant to an analysis of alternative explanations of the effects, which is needed to provide evidence for a particular TM.

**Conclusions:**

Research on psychotherapy needs to be refocused from treatment packages (TP) to treatment models (TM). This requires an improved conceptualization of the methodological principles and skills involved, and the development of valid measures of these, but also improved reporting standards concerning treatment-construct validity in scientific journals.

**Electronic supplementary material:**

The online version of this article (doi:10.1186/s40359-016-0151-2) contains supplementary material, which is available to authorized users.

## Background

Consider the following example: A specific form of manualized psychotherapy, let us call it ABC therapy, is tested in a randomized controlled trial (RCT) with depressed patients and is found to reduce depression more than a waiting list control group. Now, these results can be described at a number of different abstraction levels, as for example:

1. ABC therapy as carried out by these specific therapists with this sample of patients in this context caused reductions in depression.
2. ABC therapy caused reductions in depression.
3. Psychotherapy caused reductions in depression.

What is said under description (1) is merely what was actually shown empirically. The strength of an RCT design is its internal validity. Internal validity in psychotherapy research refers to the ability to conclude that a certain treatment package (TP) as implemented in a particular context, as distinct from anything *external* to this particular TP, caused certain effects. This corresponds to the definition of internal validity as “local, molar, causal validity” given by Shadish, Cook, & Campbell [56]. This level of description may be referred to as *the TP level of description*. A TP can be defined as a set of treatment components (procedures, interventions, ways of relating to the client, etc.) and the way they are actually combined in the treatment.

What is said under description (2) is quite compatible with the empirical results but goes clearly beyond these, as it implies *an attribution of the effects to a particular treatment model* (TM): the ABC model of therapy as conceptualized in the literature by its developers. That is, it invokes construct validity in addition to internal validity. This level of description may be referred to as *the TM level of description*. A TM can be defined as a set of hypotheses about how a certain set of treatment components (procedures, interventions, ways of relating to the client, etc.) contribute causally to certain kinds of effects in the client.

What is said under description (3) is likewise quite compatible with the empirical results, but just as description (2) it also goes beyond these – in this case by attributing the effects to psychotherapy in general. It differs from description (2) by not taking the conceptual model for granted that was used by the therapists who developed ABC therapy. That is, it implies an alternative TM of the effects shown by ABC therapy, attributing the effects of the treatment to factors that are common to what Wampold et al. [64] have referred to as forms of “bona fide” psychotherapy. What characterizes all “bona fide treatments”, according to these authors, is that they involve a theoretical rationale based on psychological principles which are available in the form of professional books or manuals, and are carried out by trained therapists who believe in, and are loyal to the given form of treatment.

Strictly speaking, this means that in our ABC therapy example there is a logical gap between the TP level of description of the effects, with its emphasis on internal validity, and the TM level of description, with two alternative *attributions of the effects to different constructs*. Although these two competing attributions are equivalent with regard to the results from singular RCTs, they are not equivalent with regard to the whole set of possible RCTs. To decide between these two alternative attributions of the treatment effects, ABC therapy may be compared with other forms of “bona fide” psychotherapy. If these comparisons find that ABC therapy is superior to other forms of psychotherapy, it is evidence for attributing the effects to ABC therapy. But if such comparisons show no significant differences in efficacy, this is evidence for attributing the effects to some kind of “common factors”.

But a number of other causal attributions are also possible. Maybe it was not even *psychotherapy* that caused the effects in the ABC therapy trial, but something that psychotherapy *shares* with a number of other procedures? For example, attributions of the effects to “having the opportunity to talk to a supportive person” (whether that person is a trained psychotherapist or not) or to “undergoing a credible treatment procedure” (even if that procedure primary involves non-verbal activities, like physical exercise) are equivalent with regard to the results from this single RCT. To rule out these explanations, and obtain evidence that psychotherapy has an effect on depression, psychotherapy has to be shown to be more effective than support from a paraprofessional, and more effective than physical exercise, respectively (or, alternatively that different mechanisms are involved even in the case of equivalent effect sizes).

Although leading methodologists like Kazdin [27] and Shadish et al. [56] are very explicit about the differentiation between internal validity and construct validity, this distinction does not always seem to be well understood among psychotherapy researchers. By controlling factors *outside of* therapy through randomization, an experimental design maximizes internal validity, and thereby helps showing that the documented effects are the result of a particular TP as it was implemented in a particular context. This inference, however, is not only “local” but also “molar”; that is, it applies to *the entire treatment package and its implementation*, and can say nothing about *what* it was about this particular treatment that was causally responsible for these effects. That is, even if an RCT is characterized by strong internal validity, this provides no evidence for a *treatment model*. It is important to remember that, whereas internal validity depends on how well a certain study is able to control for potential causal factors *external to* the TP (i.e., personal characteristics of the patients, and external events occurring concurrently with the treatment), construct validity here depends on how well it is able to rule out alternative explanations referring to other potential causal factors *within* the TP (i.e., other than those specified by the TM).

The last decades have seen important improvements in the reporting standards required of journal articles. In this context, however, it is interesting that, although the JARS (Journal Article Reporting Standards) that are included in the APA manual [2] require authors to discuss threats to internal validity and external validity (generalizability), nothing is mentioned of the need for an explicit discussion of threats to construct validity (i.e., alternative explanations concerning the active ingredients of the TP).

The question about what causes change in psychotherapy is possibly the most difficult question in psychotherapy research. Our knowledge in this area is still quite limited – as summarized by Kazdin [29], “after decades of psychotherapy research we cannot provide an evidence-based explanation for how or why even our most well-studied interventions produce change” (p. 426). Kazdin’s main focus, however, is on the development of knowledge about *mechanisms* of therapeutic change, rather than on the therapeutic *components* that contribute to change. To search for critical components is not to look for mechanisms, because “[a] component might achieve its effects for all sorts of reasons (processes) that must be assessed” ([29], p. 11). Yet, it may be argued that knowledge about critical components is extremely important in itself – for example, it may help focus the training of psychotherapists on the skills that are the most important for therapeutic change to occur. To reiterate: treatment components are therapist actions and other controllable aspects of a treatment, whereas treatment mechanisms are processes whereby therapist actions cause change in the patient. The focus here is on components, not on mechanisms.

In the present paper some steps are taken towards the development of a model for how to analyze alternative explanations in psychotherapy research. This means that the focus is on what is traditionally referred to as “construct validity”, but in particular a certain subcategory that may be referred to as *treatment-construct validity* – that is, the constructs that are used to describe the *treatment* and its active ingredients, and other alternative constructs that provide alternative explanations for its effects. To approach these questions the present paper first introduces the concept of *treatment contrasts*, and then goes on to list a variety of treatment contrasts that may be relevant to the understanding of what is causally effective in psychotherapy, with a focus on the treatment of borderline personality disorder (BPD). The basic idea of an analysis of treatment contrasts is then illustrated by applying it to a set of existing RCT studies of the treatment of BPD, for the purpose of analyzing the extent to which published RCT studies in this area provide data that make such an analysis feasible.

### The analysis of treatment contrasts

A *treatment contrast* is defined as a contrast between two TPs that may be potentially important for treatment outcome. What is contrasted by the experimental design in a comparative RCT study are two or more types of TPs as labeled according to their theoretical origin (e.g., a form of cognitive-behavior therapy and a form of psychodynamic therapy). But these TPs may also differ on a number of other dimensions. Examples are differences in therapist factors (experience, competence, particular skills, etc.), dosage (number of sessions, length of sessions, etc.), consistency and credibility of the treatment (the existence of a clear theoretical rationale for the treatment, etc.), supervision arrangements, the use of non-specific relational factors (empathy, validation, support, etc.), and the use of medication in addition to psychological treatment. Researcher allegiance also represents a potential treatment contrast, to the extent that the researchers’ beliefs and interests affect the methodological quality of how the TPs are implemented.

As long as these variables are not controlled they pose a threat to treatment-construct validity. That is, if treatment X is found to be superior to treatment Y, and treatment X also contains more than treatment Y of any of the other above-mentioned factors (i.e., more competent therapists, more therapy sessions, more consistency, a more credible theoretical rationale, more supervision, a more supportive, empathic and validating therapeutic style, more of medication, or researcher allegiance in favor of X), then these contrasts represent alternative theoretical explanations of the superior efficacy of treatment X.

The analysis of treatment contrasts is of most interest when two or more well-defined treatments are compared. RCTs that compare a well-defined treatment with a waiting list control group have minimal treatment-construct validity, because an outcome in favor of the active treatment is compatible with a large number of different explanations (e.g., being listened to by a professional therapist, undergoing a treatment procedure in general, getting new perspectives on one’s problems, etc.). Treatment as usual (TAU) may be a better comparison for pragmatic reasons, because a demonstration that a new treatment is more effective than a genuine form of TAU (i.e., a TAU that is truly representative for actual treatment as usual) indicates that clinical practice may be improved by the implementation of this treatment. For such a comparison to be of theoretical interest, however, TAU should be specified in detail, in terms of what was actually done during the treatment, to eliminate as many potentially important alternative explanations as possible (cf. [65]). Often, a TAU condition may include a mix, where only a subgroup of the patients did receive psychotherapy. The more of psychological treatment that is included in a TAU control condition, the more interesting conclusions may be drawn from its results.

In some cases, TAU actually means the absence of psychological treatment. For example, the first controlled trial of Mentalization-Based Treatment (MBT) for BPD [8] compared MBT with a form of TAU that included standard psychiatric care with no formal psychotherapy. The explicit purpose was merely to control for spontaneous remission. Although the positive results for MBT in that study are consistent with the specific TM that underlies MBT, they are also consistent with a wide variety of other possible explanations. For example, they are consistent with the hypotheses that all credible, theoretically based treatments that have been developed specifically for BPD are equally effective, or that simply having a professional person to talk to regularly during a certain period of time is better than having no such person to talk to. In other words, this study is not able to eliminate many alternative explanations, and has low treatment-construct validity.

In other cases, TAU does include psychological treatment. For example, in the first RCT with Dialectical Behavior Therapy (DBT), Linehan et al. [37] randomized the patients either to DBT or to a TAU condition where they were offered alternative therapy referrals, from which the patients could choose. As a result, 16 of the 22 patients in the control condition underwent individual therapy, whereas six did not. Although this TAU condition controls for more than spontaneous remission, and has slightly higher construct validity than Bateman and Fonagy’s [8] first MBT study, still the positive results for DBT in that study are also consistent with a large variety of possible explanations, and are difficult to use for theoretical purposes.

Treatment contrasts can be categorized as experimental or alternative. An example of an *experimental* contrast is that between DBT and Transference-Focused Psychotherapy (TFP) in Clarkin et al.'s [16] study. Here two TPs based on different theoretical assumptions are contrasted by an experimental design. To demonstrate experimental treatment contrasts of this kind, data on *treatment integrity* (defined as the extent to which the TP is implemented as intended) are needed. All other dimensions on which two TPs may be contrasted, and which thereby pose a threat to the construct validity of the conclusions, are referred to here as *alternative* contrasts.

#### Treatment integrity

Treatment integrity is defined by Perepletchikova, Treat and Kazdin [28] as the extent to which a treatment package is implemented as intended, and has three aspects: (a) therapist adherence (i.e., the degree to which the therapist utilizes prescribed procedures and avoids proscribed procedures); (b) therapist specific competence (i.e., the level of the therapist’s skill and judgment in carrying out this particular treatment); (c) and treatment differentiation (i.e., whether the TPs that are being compared differ from each other along critical dimensions).

Different forms of psychotherapy differ in their theoretical hypotheses about what makes the treatment work, and what has to be included in the TP for it to count as an example of that specific form of therapy. With regard to BPD treatments, for example, there are at least four different TMs that have been tested in RCTs with some success: DBT [35], MBT [9], TFP [17] and Schema-Focused Therapy (SFT; [66]). These four TMs clearly describe different processes that are assumed to account for the effects of treatment. The empirical presence of such DBT-, MBT-, TFP- and SFT-specific processes in a treatment condition, and the empirical absence of other processes that do not belong to the specific TM, is a matter of *treatment integrity*.

In addition to these theoretically specific experimental contrasts, the implementation of the TPs may also differ on a number of other factors. The following list includes a number of alternative treatment contrasts, but makes no pretension of being complete.

#### The therapist factor

Evidence indicates that therapists differ in terms of the outcome they achieve with their patients. The size of this therapist factor varies considerably between different studies, but in a recent meta-analysis [6] 5 % of the variability in outcome was due to the therapist factor. This poses a threat to the construct validity of the conclusions that are drawn from an RCT that compares two different treatment models – for example, if one treatment is associated with a better outcome than another, this might be due to the therapists involved rather than to the treatment method. There are in principle two possible ways of trying to eliminate the therapist factor by choice of design: (a) by randomizing therapists to the TPs that are to be compared, or (b) by using the same therapists in both TPs. In research on the treatment of BPD, the former option was used by Bateman and Fonagy [10], and the latter by Turner [59, 60]. Both options, however, may cause problems if there is therapist allegiance for one TM over another (Falkenström et al. [20]. Other possibilities are to match the therapists in terms of competence or experience, and/or to check afterwards for possible differences in therapeutic skills, abilities and experience.

#### Dosage

Treatments may differ in dosage, defined as the number of sessions or the length of sessions. This may occur either by design (i.e., one form of treatment being longer or more intensive than another) or because of more absence or dropout in one treatment than in another. Correlational evidence suggests that there is at least a weak dose-effect relationship in psychotherapy (e.g., [49]), and Howard et al. [26] suggested that this dose-effect relationship can best be characterized as negatively accelerating (i.e., with each successive session having less impact on a patient’s well-being). Consistent with this reasoning, Lambert [31] reports evidence of a dose-effect relationship across five studies, and a tendency for the effect to flatten as the number of sessions increase.

#### Consistency

A “common factor” which has been strongly emphasized by many writers, starting with Rosenzweig [55], is the consistent use of a theoretical rationale throughout the treatment. Frank and Frank [21] argued that, although the conceptual perspectives offered by different forms of psychotherapy vary widely, the important thing is that they are able to provide a plausible explanation for the client’s problems, guide the client through a therapeutic procedure based on this conceptualization, and thereby help him or her to develop new perspectives on life. A similar theme is central to Wampold et al.’s [64] notion that all “bona fide psychotherapies” are equally effective. With regard specifically to the treatment of personality disorders, Livesley [40] argues that the treatment environment has “a substantial impact because, in most settings, patients have contact with several professionals, creating opportunities for confusion and inconsistency. These problems can only be avoided if all involved in a patient’s care follow a treatment plan.” (p. 445). Regular supervision is also considered especially important when working with BPD patients. With regard to the treatment of BPD, *the provision of a borderline-specific rationale* for the treatment is an essential part of consistency.

#### An empathic, validating and supportive therapeutic stance

Empathy, warmth, and an unconditional positive regard were given a central role in psychotherapy by Rogers [53], and meta-analyses show a moderately strong association between empathy and therapy outcome [19]. With regard to the treatment of personality disorders in particular, Livesley [40] argues that the most appropriate stance is to “provide support, empathy, and validation” (p. 443). A number of psychodynamic therapists (for an overview see [5]) have also argued for the importance of a warm, human, benevolent and supportive therapeutic attitude in the treatment of BPD. The central importance of empathy and validation in treating BPD patients is similarly emphasized in Linehan’s [35, 36] writings on DBT and by psychodynamic therapists such as Gunderson and Links [24]. As Livesley [40] describes it,

“Validating responses have multiple functions. They are inherently empathic and supportive and, hence, strengthen the alliance. Recognizing, acknowledging, and accepting the effects of adverse experiences also have a settling effect early in treatment, when the search for acceptance and understanding is often a major component of crisis behaviour. Consistent validation helps to counter earlier invalidating experiences and thereby promotes self-validation and the development of a more adaptive self-structure” (p. 445–446).

#### Medication

Symptom-targeted medication management is a commonly recommended practice in the treatment of BPD (e.g., [1]), and is seldom controlled as part of the experimental design in RCT studies of psychotherapy with BPD patients. It is therefore a possible threat to the construct validity of the conclusions that need to be taken account of.

#### Researcher allegiance

Researcher allegiance (RA), defined as the researcher’s preference for a particular treatment, has been claimed to be a strong determinant of outcome in clinical trials that compare two psychological treatments (e.g., [41, 63]). A correlation between RA and treatment outcome does not in itself show anything about the direction of causality (e.g., [34]) – RA in favor of one treatment might, in fact, appear as a natural result of outcome research which has shown this form of treatment to be more effective. Munder et al. [48], however, in a meta-analysis of 79 direct comparisons from 48 treatment studies of depression and PTSD, reported evidence that RA is more strongly associated with outcome when the methodological quality of the study is low. Their results suggest that RA may lead to methodological weaknesses in the comparison conditions, and thereby cause biased results. For example, researcher enthusiasm for one particular treatment may lead to different levels in the therapists’ commitment to the two treatments that are compared, and to differences in the quality of the implementation of the two treatments. Munder et al. [48] also found that differences in the conceptual quality of the treatments (defined in terms of Wampold’s criteria for bona fide psychotherapy) mediated the RA-outcome associations – that is, researchers with a clear preference for one treatment were more likely to choose a less credible comparative treatment as control condition than researchers with more balanced preferences.

#### Measuring treatment-construct validity

In principle, it should be possible to measure the degree of treatment-construct validity in an RCT by measuring treatment integrity and other alternative treatment contrasts. Maximal construct validity would require that an RCT is designed so that (1) the treatment packages that are compared can be clearly contrasted in terms of treatment integrity, and (2) alternative treatment contrasts can be eliminated. Construct validity is threatened when there is (1) insufficient treatment integrity, or insufficient data on treatment integrity (i.e., a lack of data on adherence, competence and differentiation between the treatments), or (2) an absence of data on alternative treatment contrasts, or data that show such contrasts between the TPs. The more such threats to construct validity that can be eliminated, the higher is the construct validity of the conclusions that can be drawn from a study.

In the next part of the present paper this kind of analysis is applied to comparative RCTs of psychotherapy with patients diagnosed with BPD. The main purpose here is to explore to what degree published studies in this area allow conclusions concerning possible alternative explanations of the results, and if they differ in this regard in a way that could make it possible to rank order RCTs in terms of treatment-construct validity.

## Method

A systematic search of the literature was done to find studies of the treatment of Borderline Personality Disorder published until 2014, which (1) used an RCT design, (2) compared two or more psychotherapy conditions, (3) included at least 10 patients in each condition, (4) where the majority of patients engaged in self-harm before treatment, and (5) self-harm (suicidal and/or non-suicidal) was among the outcome measures. For this purpose we used online databases (PubMed, PsycINFO, Medline), starting with a broad search which combined the terms “Borderline personality disorder”, “treatment” and “random*”, searching for studies which satisfied the above-mentioned inclusion criteria. This resulted in the identification of eight trials, as summarized in Table 1. Because information from several of these trials were reported not only in the primary study mentioned in Table 1 but also in a series of secondary studies, we chose to refer to these trials primarily in terms of the treatments contrasted (e.g., DBT-o vs. CCT), rather than by referring to singular published studies. The reporting of these studies is made in accordance with PRISMA guidelines [46]. To increase transparency, more detailed information about the coding of these studies is available in an Additional file 1 titled “Codings of eight RCTs comparing different forms of psychotherapy for Borderline Personality Disorder”.

**Table 1:** Descriptive data on the eight comparative RTCs included in the analysis

Primary study	Treatments compared	No of patients	Outcome
Turner (2000) [59]	DBT-o vs. CCT	24	DBT-o > CCT
Linehan et al. (2002) [39]	DBT vs. CVT + 12S	23	No sign diff
Giesen-Bloo et al. (2006) [22]	SFT vs. TFP	88	SFT > TFP
Linehan et al. (2006) [38]	DBT vs. CTBE	101	DBT > CTBE
Clarkin et al. (2007) [16]	TFP vs. DBT vs. SPT	90	No sign diff
Bateman & Fonagy (2009) [10]	MBT vs. SCM	134	MBT > SCM
McMain et al. (2009) [44]	DBT vs. GPM	180	No sign diff
Doering et al. (2010) [18]	TFP vs. Exp	104	TFP > exp

*CCT* Client-Centered Therapy, according to Carkhuff et al.’s [15] manual

*CTBE* Community Treatment by Experts (nominated by community mental health leaders as being especially skillful in treating difficult clients; [38])

*CVT-12S* Comprehensive Validation Therapy (the acceptance/validation part of DBT), in combination with a 12 step Narcotics Anonymous program

*DBT* Dialectical Behavior Therapy [35]

*DBT-o* DBT-oriented therapy, a modified form of DBT [59, 60]

*Exp* Experienced community psychotherapists (mainly psychoanalysts and behavior therapists; [18])

*GPM* General Psychiatric Management (including psychodynamic therapy according to [24])

*MBT* Mentalization-Based Treatment [9]

*SCM* Structural Clinical Management (Bateman, A., Fonagy, P., Bolton, R., & Karas, E: Structured clinical management for borderline personality disorder, unpublished)

*SFT* Schema-Focused Therapy [4, 66]

*SPT* Supportive Psychodynamic Therapy [3, 52]

*TFP* Transference-Focused Psychotherapy [17]

The treatment conditions in these studies are either clearly defined forms of psychotherapy or involve “expert therapists” [38] or experienced community therapists [18]. The two latter studies used therapists who were recruited as being especially skillful and interested in the treatment of BPD patients. The reason to include the two latter treatment conditions, despite the fact that the actual therapies in that condition were not homogenous, is that the treatment in both cases were carried out by qualified psychotherapists who were either categorized as “expert” or as highly experienced (which according to some theories are sufficient for therapy to work), and who also had access to regular supervision.

### The coding of treatment contrasts

#### Experimental contrasts

Experimental contrasts were coded in terms of the labels of the treatment conditions (DBT, TFP, MBT, SFT, etc.). For each RCT comparison, a *treatment integrity index* (TII) was computed on the basis of whether (1) the treatments were monitored for adherence by supervisors, (2) measures were used demonstrating good adherence, (3) measures were used demonstrating good competence, and (4) measures were used demonstrating good differentiation. Each item was coded either as 1 (if this was true for both TPs) or as 0 (if this was not true for both TPs). The scores were added and divided by 4, resulting in a TII that may range from 0 to 1.

### Alternative contrasts

Alternative contrasts were coded in terms of three broad alternatives: (1) Data reported show a difference between the two TPs. (2) Data reported show no evidence of a difference between the two TPs. (3) No data are reported. For each RCT an *alternative treatment contrast index* (ATCI) was computed, defined as the number of alternative treatment contrasts that were coded as “no evidence of a difference” between the treatments, and dividing this with the total number of potential factors that were defined a priori. This means that the ATCI can range from 0 to 1. The following alternative treatment contrasts were coded:

*The therapist factor* was coded in terms of quantitative data on therapists’ years of clinical experience (because this was the only commonly available kind of data), and was concluded to differ if the therapists in one of the treatment conditions had significantly more clinical experience than the therapists in the other treatment condition. When no statistical comparison was made on this factor, it was coded as “no data reported”.

*Dosage* was measured by the number and length of treatment sessions reported in the studies, and was coded as different if the patients in one of the treatment conditions received significantly more therapy time than patients in another treatment condition.

*Supervision* was coded in terms of data on the frequency and duration of supervision, and was coded as different if the therapists in one treatment condition received more supervision than the therapists in another treatment condition.

*Borderline-specific rationale* (as an operationalization of consistency) was coded as positive if a treatment used a BPD-specific manual based on an explicit theory about the etiology and treatment of BPD. The treatments were coded to differ on this factor if only one of them was based on such a BPD-specific rationale.

*An empathic, validating and supportive therapeutic stance* was coded on the basis of (1) the priorities formulated in the treatment manual, and (2) patients’ ratings of the therapist’s stance (including the therapist’s contribution to the working alliance). This factor was coded as different if there was an obvious difference in the priorities formulated in the treatment manual (i.e., so that the emphasis on an empathic, supportive and/or validating stance is more emphasized in one treatment than in the other) and/or if the patients rated one treatment higher than the other on a measure of the therapist’s contribution to the working alliance or some similar measure.

*Medication* was coded as different if the number of patients who were on medication during treatment differed significantly between the conditions.

*Researcher allegiance,* defined as the researcher’s preference for a particular treatment, was rated in terms of the three direct indicators used by Munder et al. [48] in their meta-analysis of RA: author developed the treatment, author advocates the treatment, and author has contributed to an etiological model which is consistent with the treatment. Allegiance was coded as being in favor of one treatment condition if a larger number of indicators favored this treatment than the other.

### Procedure

The coding was made independently by the three authors, who have different theoretical orientations (integrative, psychodynamic, and cognitive-behavioral). When some factor was coded differently, this was discussed until consensus was reached. For some discrepancies, this only required a closer reading of passages in the available text. For a few discrepancies, however, consensus could be reached first after more elaborate discussion.

## Results

As seen in Table 1, the eight RCTs varied both in sample size and clinical outcome. In five of the studies one treatment was superior to another; whereas in three studies there was no significant difference. In total, these studies included ten clearly specified forms of treatment, of which at least seven (DBT, GPM, MBT, SFT, SPT, and TFP) can be classified as “bona fide”, in the sense that they involved a theoretical rationale based on psychological principles which was available in the form of professional books or manuals, and were carried out by trained therapists with an allegiance to the given form of treatment. Yet another treatment (CCT) was clearly based on psychological principles and described in a manual, although it is unclear to what extent the therapists had an allegiance to the model in this case (because the same therapists carried out both TPs that were compared). Two other of the TPs (DBT-o and CVT + 12S) were derived from DBT and were constructed for that particular study; and still another one (SCM) was constructed specifically for the particular study without being based on any clear theoretical rationale.

The results on treatment integrity are summarized in Table 2, and the analysis of alternative treatment contrasts is summarized in Table 3. Short summaries of these analyses are given below for each of the eight RCT studies; more detailed information about the treatments and the codings of outcome, treatment integrity and alternative treatment contrasts is found in the Additional file 1 “Codings of eight RCTs comparing different forms of psychotherapy for Borderline Personality Disorder”.

**Table 2:** Treatment integrity as assessed in eight RCTs which compare different forms of psychological treatments for Borderline Personality Disorder

	Adherence monitored by supervisors	Evidence of adherence	Evidence of competence	Evidence of differentiation	Treatment Integrity Index (TII)
DBT-o vs. CCT	1	0	0	0	.25
DBT vs. CVT + 12S	1	0	0	0	.25
SFT vs. TFP	1	1	0	1	.75
DBT vs. CTBE	0	0	0	0	.00
TFP vs. DBT vs. SPT	1	0	0	0	.25
MBT vs. SCM	1	1	0	0	.50
DBT vs. GPM	1	1	0	1	.75
TFP vs. Exp	0	0	0	0	.00

1 = true for both TPs; 0 = not true for both TPs. The scores for each item were added and divided by 4, resulting in a TII that may range from 0 to 1

*CCT* Client-Centered Therapy, *CTBE* Community Treatment by Experts, *CVT-12S* Comprehensive Validation Therapy combined with a 12 step program, *DBT* Dialectical Behavior Therapy, *DBT-o* DBT-oriented therapy, a modified form of DBT, *Exp* Experienced community psychotherapists, *GPM* General Psychiatric Management, *MBT* Mentalization-Based Treatment, *SCM* Structural Clinical Management, *SFT* Schema-Focused Therapy, *SPT* Supportive Psychodynamic Therapy, *TFP* Transference-Focused Psychotherapy

**Table 3:** The analysis of alternative treatment contrasts in eight comparative RCTs of treatments for Borderline Personality Disorder

Treatment contrast	DBT-o vs. CCT	DBT vs. CVT	SFT vs. TFP	DBT vs. CTBE	TFP vs DBT vs. SPT	MBT vs. SCM	DBT vs. GPM	TFP vs. Exp.
Therapist experience	0	-	0	1	-	0	0	0
Dosage	0	1	-	1	-	0	1	1
Supervision	0	0	0	1	0	0	0	1
BPD-specific rationale	1	0	0	-	0	1	0	-
Empathy, validation, support	0	0	1	1	1	0	0	-
Medication	-	0	0	1	0	1	0	0
Researcher allegiance	1	1	1	1	1	1	0	1
Number of eliminated contrasts	4	4	4	0	3	4	6	2
ATCI	.57	.57	.57	.00	.43	.57	.86	.29

0 = no evidence of a difference; 1 = evidence of a difference; - = no data reported

*ATCI* Alternative Treatment Contrast Index

*CCT* Client-Centered Therapy, *CTBE* Community Treatment by Experts, *CVT-12S* Comprehensive Validation Therapy combined with a 12 step program, *DBT* Dialectical Behavior Therapy, *DBT-o* DBT-oriented therapy, a modified form of DBT, *Exp* Experienced community psychotherapists, *GPM* General Psychiatric Management, *MBT* Mentalization-Based Treatment, *SCM* Structural Clinical Management; *SFT* Schema-Focused Therapy, *SPT* Supportive Psychodynamic Therapy, *TFP* Transference-Focused Psychotherapy

### The eight studies

#### DBT-oriented therapy vs. Client-Centered Therapy [59]

Although two supervisors monitored adherence to the respective treatment protocols, no data are reported on adherence, competence, or differentiation, thereby producing a TTI of .25. As seen in Table 3, four of the seven alternative treatment contrasts (therapist experience, dosage, supervision, and empathy/validation/support) showed no evidence of a difference, thereby producing an ATCI of .57. Apart from the experimental contrast (i.e., DBT-o vs. CCT), this leaves at least two alternative contrasts as possibly contributing to the superior effects of DBT-o: (1) the use of a clear BPD-specific rationale, and (2) a researchers’ allegiance in favor of DBT-o.

#### Study 2. DBT vs. Comprehensive Validation Therapy [39]

Although therapists in each condition met weekly with supervisors to discuss case material and review session videotapes to promote adherence to treatment manuals, no data on adherence, competence, or differentiation were reported, resulting in a TTI of .25. As seen in Table 3, this study apparently managed to eliminate four of seven treatment contrasts (BPD-specific rationale, supervision, empathy/validation/support, and medication), rendering it an ATCI of .57. Although the dosage and allegiance factors were in favor of DBT, the treatments did not differ significantly in efficacy.

#### Study 3. SFT vs. TFP [22, 58]

Treatment integrity was monitored by means of supervision, and assessed by other therapists who rated the adherence and competence on specifically developed scales with an identical cutoff score of at least 60. The results showed clear evidence of adherence and differentiation. In terms of differentiation, a psychologist who was blind to allocation listened to one randomly selected taped session from each patient, and was able to correctly classify 85 of 86 tapes ([22], p. 651). Although competence was rated as satisfactory for both treatments, the higher competence ratings for SFT (73) than for TFP (60) represent a possible threat to treatment-construct validity, rendering a less than optimal treatment integrity index (TTI = 0.75). As seen in Table 3, four of seven alternative contrasts (therapist experience, a BPD-specific rationale, supervision and medication) showed no evidence of a difference, resulting in an ATCI of .57. Remaining as potential contributing factors to the superior outcome of SFT were, apart from the experimental contrast (SFT vs. TFP), differences in therapist competence, a larger use of support and validation in SFT, and a researchers’ allegiance in favor of SFT.

#### Study 4. DBT versus CTBE (community treatment by experts) [11, 38]

The treatment in the CTBE condition was uncontrolled by the research team, which means that no data on treatment differentiation were reported (TTI = .00). As seen in Table 3, all analyses of treatment contrasts showed evidence of differences between the treatments, producing an ATCI of .00. Two of the factors, however, differed in the opposite direction to treatment outcome (therapist experience and medication), thereby making these factors unlikely to be causally involved in the outcome. Remaining as potential causal factors, apart from the experimental contrast (DBT vs. CTBE), were dosage, supervision, BPD-specific rationale (which, however, could not be supported by the data), degree of empathy/support/validation, and a researchers’ allegiance for DBT.

#### Study 5. TFP vs. DBT vs. SPT [16, 33]

All therapists attended weekly group supervision where they were provided feedback on the basis of videotaped sessions. Further, additional individual supervision was provided when adherence or competence fell below acceptable levels, and when a therapist fell below acceptable levels no new cases were assigned to them. No data on adherence, competence, or differentiation, however, are reported, resulting in a TTI of .25. As seen in Table 3, three of the seven alternative treatment contrasts (BPD-specific rationale, supervision, and medication) were coded as “no evidence of a difference”, which resulted in an ATCI of .43. Two other factors (empathy/support/validation and allegiance) were coded as different, although in opposite directions: more focus on empathy, support and validation in DBT and SPT, and an allegiance in favor of TFP.

#### Study 6. MBT vs. Structural Clinical Management [10]

Although data showed 85 % adherence to the MBT manual and 96 % adherence to the SCM manual, no data were reported on competence or differentiation, resulting in a TTI of .50. As seen in Table 3, this study showed no evidence of a difference on four of the seven alternative contrasts (therapist experience, dosage, supervision, and empathy/validation/support), rendering an ATCI of .57. Remaining as possible contributing factors to the superior outcome of MBT, apart from the experimental contrast (MBT vs. SCM), were two alternative contrasts: the BPD-specific rationale in MBT, and a researchers’ allegiance in favor of MBT.

#### Study 7. DBT vs. General Psychiatric Management [44, 45]

Modality-specific adherence scales were used to evaluate treatment integrity, and adherence was supported for both conditions, as well as differentiation between the treatments. However, no data were reported on competence, rendering a TTI of .75. As seen in Table 3, this study apparently managed to eliminate six of seven alternative treatment contrasts (therapist experience, BPD-specific rationale, supervision, empathy/support/validation, medication, and researchers’ allegiance), resulting in an ATCI of .86. The two TPs differed in terms of dosage (i.e., the DBT patients received more therapy), but this apparently was of no importance, as the treatments were equivalent in efficacy.

#### Study 8. TFP vs. experienced therapists [18]

No integrity checks were performed of therapies in the control condition, resulting in a TTI of .00. As seen in Table 3, two of the seven alternative contrasts (therapist experience and medication) were coded as “no evidence of a difference”, resulting in an ATCI of .29. Four other factors (dosage, a BPD-specific rationale, supervision, and allegiance) remained as possibly contributing to the superior outcome of TFP.

### Treatment integrity

As seen in Table 2, most of the studies showed rather low treatment integrity. Although adherence was systematically monitored in six of eight studies, only three of these reported quantitative data which showed adherence, and only two of these showed clear evidence of differentiation (the SFT vs. TFP trial, and the DBT vs. GPM trial). With regard to competence, only one study (the SFT vs. TFP trial) reported data, but because the competence ratings were not equivalent optimal treatment integrity (1.00) could not be assigned even to this study.

### Alternative treatment contrasts

Similar considerations apply to the measurement of alternative treatment contrasts: there is an absence of data on many variables, and even when there are data these are often of questionable quality. For example, despite the widespread assumption (e.g., [40]) that a therapeutic stance characterized by empathy, validation and support is especially important in the treatment of BPD, only three of the eight RCTs included empirical data relevant to this topic. The results show a clear differentiation between the RCTs in terms of their degree of treatment construct-validity. At the lower end (i.e., low on both TII and ATCI) is the comparison between DBT and “community treatment by experts” (CTBE). At the opposite end of the scale we find the comparison between DBT and General Psychiatric Management (GPM), which showed the highest ATCI (.75) and shared the highest TII (.75) of the eight studies reviewed. Here two TPs are compared which are clearly differentiated in terms of treatment content; and although they differed in terms of dosage (i.e., the DBT patients received more therapy), otherwise they did not appear to differ in terms of the treatment contrasts that were analyzed. Even here, however, there are a number of limitations. For example, although empathy and validation were explicitly described as primary strategies in both conditions, no measures were taken of how the patients perceived their therapists’ degree of empathy, support or validation.

## Discussion

The present study applied the analysis of treatment contrasts to eight RCTs that compare different forms of psychotherapy for BPD, most of which are published in prestigious scientific journals. The results showed that these RCTs vary widely in treatment-construct validity, and that it is difficult to draw any conclusions from these trials about what makes treatment of BPD effective. The results indicate that the publication policies of scientific journals in this area have seldom required systematic data relevant to an analysis of alternative explanations of the effects, which is needed to provide evidence for a particular treatment model.

Major gaps in data were found with regard to both treatment integrity and alternative treatment contrasts. In terms of treatment integrity (a) evidence of therapist adherence was reported only by three of eight studies (although supervision to achieve adherence was reported by most of the studies), (b) measurement of therapist competence was accomplished by only one study (which, interestingly, did *not* show equal competence between the therapists in the two treatment conditions, thereby further emphasizing the importance of assessing this variable), and (c) clear empirical differentiation of treatments was only accomplished in two studies.

In terms of alternative treatment contrasts, it is interesting to note that the eight studies showed a wide variation in their ability to eliminate possible alternative explanations, from the most well-controlled (the DBT vs. GPM study) to the least controlled ones (the two studies which compared DBT and TFP, respectively, with expert therapists). The quality of the data needed to eliminate alternative explanations was generally low.

For example, the only available data on the therapist factor was therapists’ years of clinical experience. This may be criticized as probably not being a valid indicator of therapist competence; in fact, years of clinical experience has not been shown to be reliably associated with treatment outcome in previous research (e.g., [32]). Against this background, it is curious that these are the only data generally reported on the therapist factor. This is reminiscent of the “streetlight effect”, that is, when people look for what they are searching for only where it is easiest (i.e., where there is light) – even when it is highly unlikely that something will be found there. It is easy to collect data on therapists’ years of clinical experience – therefore this is reported, even when there is little to support that this is a valid marker of therapist competence. The importance of the therapist factor in the treatment of BPD cannot be judged on the basis of this kind of data. On the other hand, we do not yet have any well-developed conceptualization of the skills and other personal characteristics that are involved in being an efficient therapist. What is required here is a well-developed conceptualization of the skills and other personal characteristics that are involved in being an efficient therapist, and the development of valid measures of these skills and characteristics.

In the absence of such measures, one may try to control the therapist factor by making use of the same therapists in the TPs that are to be compared, or by randomizing therapists across these TPs. Among the trials included in the present analysis the former was done in one (the DBT-o vs. CCT trial), and the latter in another (the MBT vs. SCM trial). Both of these strategies may cause problems if there is therapist allegiance for one TM over another; if the therapists believe more strongly in one treatment than in the other this may well affect their efficacy in carrying out these treatments. Although this may not necessarily have been a problem in the DBT-o vs. CCT trial, because the therapists are described as having “theoretical backgrounds in family-systems, client-oriented, and psychodynamic therapy” ([59], p. 415), it cannot be excluded that the therapists may have been influenced by the enthusiasm surrounding DBT when it was introduced as a new treatment for BPD in the 1990s, especially in view of the researcher’s (e.g., Turner’s) theoretical allegiance in favor of DBT [61]. Similar considerations apply to the MBT vs. SCM trial: it cannot be excluded that the therapists may have been influenced by the enthusiasm surrounding MBT when it was introduced as a new treatment for BPD in the 2000s, especially in view of the researchers’ (e.g., Bateman and Fonagy’s) theoretical allegiance in favor of MBT.

This is consistent with the conclusions drawn by Falkenström et al. [20] on the basis of a meta-analysis of 39 studies that used a crossed therapist design (i.e., when the same therapists deliver two or more forms of therapy as part of the same trial). The authors found that researcher allegiance was strongly associated with outcome in studies that did not control for therapist allegiance, and concluded that the crossed therapist design is subject to bias due to differential therapist allegiance. As they conclude, “All clinical trials, and especially crossed therapist designs, should measure psychotherapist allegiance to evaluate this possible bias” ([20], p. 482).

Another general problem is the lack of empirical data reported on some of the factors, which led us to code them largely on the basis of qualitative comparisons between treatment manuals. For example, only three of the eight RCTs included empirical data on the relational factor (i.e., degree of empathy, support and validation). In the remaining five trials, decisions about whether the treatments differed on this factor had to be based solely on design, as described in treatment manuals. The decisions made on the basis of such data led us to code the TFP manual as prescribing less of support and validation than other manuals, and to code the comparisons between the other manuals as “no evidence of a difference” on this factor. This is unsatisfactory. For example, it is not self-evident that such differences in explicit formulations between manuals correspond to analogous differences in therapists’ actual behavior in session – what would be needed here are either repeated measures of patients’ ratings of their therapists’ empathy, support and validation, or independent observers’ ratings of the degree to which the therapist actually shows empathy, support and validation in his or her way of relating to the patient.

Similarly, the decisions about whether the therapists used a consistent theoretical rationale, in terms of an etiological theory about BPD, were based solely on a comparison between treatment manuals. Again, this is unsatisfactory because it is not self-evident that the therapist’s actual behavior in session will reflect the explicit formulations in the manual. Although ratings of adherence and competence may be informative about the degree to which the therapist adheres to the manual and does so in a competent way, such ratings are not necessarily informative about the *consistency* in the therapist’s way of conveying the theoretical rationale for the treatment. It would, however, be possible to obtain such data in connection with treatment integrity ratings of video-recorded sessions.

The assessment of researchers’ allegiance relied on an established procedure: the direct indicators from Munder et al.’s [48] study. In this context, it may be noted that the only study where there was no evidence of researchers’ allegiance (the DBT vs. GPM trial; [44, 45]) found no tendency to any differences in outcome – despite the fact that this, being the largest study that has so far been carried out in this area, had a comparably high power for the detection of any real differences in outcome, and also showed relatively high treatment-construct validity.

### Limitations

The present study suffers from a number of limitations. First, we analyzed data on only seven potentially important factors: the therapist factor, dosage, supervision arrangements, the use of a BPD-specific theoretical rationale, degree of empathy/support/validation, medication, and researchers’ allegiance. The reason for choosing these particular factors was that they have all been suggested to be important in the research literature, and that data were reported on at least some of these in the RCT studies that were analyzed. This list of factors, however, in no way pretends to capture all the potentially relevant factors, nor does it make any pretense to conceptualize and differentiate the studied factors in an optimal way. This means that even the conclusions from those RCTs that showed the highest treatment-construct validity in the present analysis remain uncertain; with a more sophisticated conceptualization of treatment contrasts these studies might well be shown to leave a large number of alternative explanations uncontrolled. Further, because there is still so little empirical evidence for what makes psychotherapy work (e.g., [28]), there is no way of knowing which of these factors are most important to control.

A second limitation concerns the categorization of treatment contrasts. For example, it is not self-evident that empathy, support and validation should be lumped together into one category, as we may well imagine therapists who are highly empathic without offering much of support or validation. Bohart and Tallman [13], for example, define empathy in psychotherapy as “having a primary intention to try to understand the client in terms of the client’s frame of reference” (p. 400), which may be conveyed in connection with a large number of different interventions (including interpretations, questions, advice, suggestions of a technique, and even confrontations), and should therefore be differentiated from support and validation. To be meaningful, however, such a differentiation requires that there are data available to rate therapists on these variables separately.

Related to this, although the calculation of the ATCI index may seem to imply that all alternative contrasts are equal in importance, no such assumption can be made. The categorization of treatment contrasts is open to revision in several ways; it is quite possible, for example, that some treatment contrasts had better be differentiated into several contrasts. The rank-order of the RCTs in the present analysis must therefore be seen as hypothetical.

A third limitation is that we analyzed only eight RCTs within a limited research area, characterized by rather strict inclusion criteria. For example, we included only RCTs that compared at least two treatment conditions (both with at least 10 patients) where all patients received psychotherapy, and where the patients had to have a BPD diagnosis and engage in self-harm. This means, for example, that comparative RCTs of BPD patients where the majority of the patients did not engage in self-harm, or had less than 10 patients in each treatment condition, were not included. On the other hand, because this is the first study to test the feasibility of an analysis of treatment contrasts, it may be argued that this limitation is necessary. The amount of data to handle was found to be very large even with the inclusion of only eight RCTs, and when the first study is carried out with a new method (i.e., the analysis of treatment contrasts in the present case) it is also important to be extra explicit about the details of this method. Eight comparative RCTs in the treatment of BPD is a suitably large sample to be used for the demonstration of the basic principles of an analysis of treatment contrasts. It would be interesting to carry out similar analyses also for comparative RCTs of patients with other psychiatric disorders (e.g., depression). Such an undertaking, however, would be of another magnitude, in view of the large number of comparative RCTs that exist in this area.

A fourth limitation is that, although a number of problems have been pointed out in the present paper as regards the theoretical conclusions that can be drawn from comparative RCTs in psychotherapy research, little in the form of concrete practical advice has been offered on how these problems can be solved. For example, no advice is offered on how therapist skillfulness and competence may be assessed in a valid way when different methods are compared. On the other hand, it may be argued that an increased understanding of the various problems involved has a value in itself, and is a prerequisite to any well-informed attempt to find practical solutions.

## Conclusions

To summarize, the present paper has addressed a neglected topic in psychotherapy research – threats to treatment-construct validity, as seen in a failure to analyze alternative explanations – and has suggested a way of addressing this problem by an analysis of treatment contrasts. The results show the potential value of such an analysis of treatment contrasts in comparative RCTs, in the sense that it makes it possible at least to rank-order RCTs in terms of their treatment-construct validity. At the same time, it also indicates that the low quality of the data relevant to such an analysis in published research (at least for RCTs comparing different treatments of BPD) makes it difficult to draw any conclusions about the treatment models involved – that is, conclusions about causality at the level of theoretical constructs.

Psychotherapy research is characterized by a general neglect of construct validity, as compared with internal and external validity. An example of this kind of neglect is that the JARS (Journal Article Reporting Standards) which are included in the APA manual [2] require authors to discuss threats to internal validity and external validity, but requires nothing similar for threats to construct validity. It bears emphasizing that the ability to draw conclusions at a theoretical level about the efficacy of a certain treatment model (TM) is a matter of good construct validity, rather than internal or external validity; and this requires the development of adequate theoretical constructs, and reliable measures of these.

As described in the introduction, maximal construct validity of the conclusions about the relative efficacy of treatments in an RCT requires (1) that the treatment packages (TPs) which are compared can be clearly contrasted in terms of treatment integrity, and (2) that alternative treatment contrasts can be eliminated. This requires the researcher not only to control but also to *measure* both treatment integrity (i.e., adherence, competence and differentiation between the treatments), and alternative treatment contrasts (e.g., therapist skills and qualities, dosage, supervision, the credibility of the therapeutic rationale, relational factors, medication, and allegiance), Although a good experimental design can contribute to control, this is not sufficient; we also need good theoretical constructs that tell us *what* to control, and good measures of these.

It is true that the role of treatment *components* can also be studied experimentally by so-called dismantling designs or additive designs (e.g., [14]). A dismantling design removes components (to see if the outcome depends on the presence of certain components), whereas an additive design adds components (to see if the outcome is improved by adding new components). Meta-analyses of such component designs indicate that, although there is so far no evidence that dismantling designs can provide increased knowledge about active treatment components, additive designs do show at least some small effect of adding new components to a treatment [12]. More principally, however, even when the RCT design takes the form of such dismantling and additive studies, it is only able to contrast two or a few treatment packages, and the same problem applies here: there will always remain a large number of treatment contrasts that are not controlled by the experimental design, and that serve as potential alternative explanations of the outcome. And, again, these potential treatment contrasts have to be measured.

In other words, these issues cannot be solved simply by improving the experimental design of an RCT. The strength of an experimental design is that it can optimize *internal* validity, defined as “local, molar causal validity” ([56], p. 54), as distinct from the *external* validity and the *construct* validity of any conclusions about causality. A perfect experimental design can, in principle, do nothing more than show that a specific treatment package (TP), as it was implemented in a specific setting with specific therapists and patients, produced effects on a specific set of measures. Establishing the external validity of conclusions about this treatment – the ability to generalize to other patients, therapists, settings and measures – requires replications of these results under other conditions [56]. And establishing the construct validity of conclusions that the effects can be attributed to processes described in a given treatment model (TM) requires the researchers to measure all relevant variables involved.

An RCT design as such cannot even guarantee that the experimental conditions (i.e., the treatment packages that were implemented) conform to the treatment models that are to be contrasted. The latter requires adequate *measures* of treatment integrity, including adherence, competence and differentiation [50]. Training the therapists in TM-specific manuals before they are allowed to take part as therapists in the actual trial does not guarantee that the treatments they carry out as part of the trial show adequate adherence, competence or differentiation. The neglect of these topics – as seen, for example in the fact that only two of the eight RCTs in the present analysis included measures of differentiation – poses a threat to the construct validity of the conclusions that can be drawn from the results.

The same goes for alternative treatment contrasts – without measuring them they cannot be eliminated as potential threats to the construct validity of the conclusions. For example, even though the experimental design may set out to control dosage or supervision arrangements by keeping them similar across treatment conditions, the *actual* dosage and the *actual* supervision received have to be measured. Even more important – but also more difficult – is the need to measure therapist factors and relational factors that have been invoked as essentially involved in the treatment especially of patients with personality disorders (e.g., [40]). What is required to rule out these alternative explanations (or at least render them unlikely) is reliable measurement of these factors, and a sophisticated analysis of the associations between these factors and outcome. Unfortunately, as the present analysis shows for RCTs of the treatment of BPD, there is a relative absence of data on these alternative treatment contrasts, which makes it difficult to draw causal conclusions from this research.

The situation would improve if more attention were paid to these kinds of data in the future design of such studies. This may, however, require the development of better measures of important variables (e.g., therapist skills and other qualities, relational factors such as empathy, support and validation, factors related to the credibility of the treatment, treatment consistency, etc.). Some of these variables may probably be measured in terms of observer ratings based on video-based recordings of sessions. Others might be measured by means of the calculation of new variables from time-series analyses; for example, Ramseyer et al. [51] used a time-series panel analysis of session-to-session aspects of change and found that therapists’ consistency over time in their use of treatment interventions (as measured by auto-correlations between adjacent sessions) was positively associated with better outcomes.

The development of better measures, however, probably also requires an improved theoretical conceptualization of the methodological principles and therapeutic skills that may be hypothesized to be important for outcome. Here it may be argued (e.g., [23, 42, 43, 54, 62]) that psychotherapy research would benefit by a shift of focus from treatment packages to a systematic specification of basic methodological principles and therapeutic skills. Ideally, this would require a comprehensive, integrative theoretical conceptualization of psychotherapy, in terms of which both common factors and more specific factors can be delineated and operationalized.

Because of the practical difficulties and large costs involved in RCTs which attempt to control all relevant variables, it may be asked what is the proper role of experimental designs in psychotherapy research, and if there are other research paradigms that may prove fruitful when it comes to prioritizing construct validity. Although experimental designs have the advantage of being able to show if a treatment is better than no treatment or than a TAU condition, such comparisons are probably important primarily for “political” purposes (as an argument that a certain form of treatment should receive public support), whereas they have so far contributed very little to the theoretical understanding of what makes psychotherapy work [29]. The use of experimental designs to compare different forms of psychotherapy has so far been unable to provide strong evidence that any one treatment model is superior to any other [63]. Consistent with this, the RCT that was ranked as highest in terms of construct validity in the present analysis (the DBT vs. GPM trial) showed no evidence whatsoever of differential efficacy. This suggests the hypothesis that the higher the treatment-construct validity, the less of a difference between different TPs will be found – a hypothesis that might be possible to test in a meta-analysis with treatment-construct validity, quantified in terms of treatment contrasts (ATCI and TII indexes), studied as moderators.

An increased focus on construct validity would mean that the development of psychotherapy theory and adequate measures of theoretical constructs should be prioritized, and this might well proceed also as part of practice-based research (e.g., [25]), and the use of repeated measures during treatment to establish the timeline [28] between different kinds of interventions (or changes in the therapeutic relationship) and psychological changes in the patient. Such a change of focus may also involve the use of a more person-oriented approach (e.g., [43]) to psychotherapy research, including various forms of single-subject designs [7, 30, 47, 57].

In addition, the reporting standards required of journal articles need to be improved, so that they require authors to discuss threats to the construct validity of the conclusions as well as threats to internal validity and external validity. It is striking that, although the JARS (Journal Article Reporting Standards) which are included in the APA manual [2] require authors to discuss threats to internal validity and external validity, nothing similar is required for threats to construct validity.

## Additional file

Additional file 1: Codings of eight RCTs comparing different forms of psychotherapy for Borderline Personality Disorder is added, which contains more detailed information about the treatments and the coding of outcome, treatment integrity and alternative treatment contrasts by the coders. (DOCX 44 kb)

## Abbreviations

ATCI: Alternative treatment contrast index
BPD: Borderline personality disorder
CCT: Client-Centered Therapy
CTBE: Community treatment by experts
CVT: Comprehensive Validation Therapy
DBT: Dialectical Behavior Therapy
GPM: General Psychiatric Management
MBT: Mentalization-Based Treatment
RCT: Randomized controlled trial
SCM: Structural Clinical Management
SFT: Schema-Focused Therapy
SPT: Supportive Psychodynamic Therapy
TFP: Transference-Focused Psychotherapy
TII: Treatment integrity index
TM: Treatment model
TP: Treatment package
