# Supplementary material for: The neglect of treatment-construct validity in psychotherapy research: a systematic review of comparative RCTs of psychotherapy for Borderline Personality Disorder
Source: BMC Psychol. 2016 Aug 24;4(1):44. doi: 10.1186/s40359-016-0151-2 (PMC4997665; doi:10.1186/s40359-016-0151-2)
Supplement: Additional file 1: — Codings of eight RCTs comparing different forms of psychotherapy for Borderline Personality Disorder is added, which contains more detailed information about the treatments and the coding of outcome, treatment integrity and alternative treatment contrasts by the coders. (DOCX 44 kb) [file 40359_2016_151_MOESM1_ESM.docx]

# Codings of eight RCTs comparing different forms of psychotherapy for Borderline Personality Disorder

# Data Supplement to the article “The neglect of construct validity in psychotherapy research: A systematic review of comparative RCTs of psychotherapy for Borderline Personality Disorder”

Lars-Gunnar Lundh, Terese Petersson, and Martin Wolgast

The eight RCT studies are summarized below and analyzed in terms of treatment integrity and alternative treatment contrasts.

**Study 1. DBT-oriented therapy vs. client-centered therapy (Turner, 2000a)**.

This study was the first to compare DBT with another form of psychotherapy: client-centered therapy (CCT), according to Carkhuff et al.’s (1976) manual. CCT was chosen as the contrasting treatment condition because it was considered to be a form of supportive therapy, and “many clinicians view supportive psychotherapy models of treatment as appropriate for BPD patients” (p. 414). This study also differed from previous research on DBT by the fact that the treatments were conducted by mental staff in a community mental health clinic, “in the context of the real-world comorbidity and complexities associated with this diagnosis” (p. 414), and by including both female and male patients. The sample, however, was small: 24 patients (19 women and 5 men). Twelve of these were randomized to one year’s treatment with a modified form of DBT (referred to as DBT-oriented therapy), and twelve to one year of CCT.

***Outcome*.** Assessment was conducted at pretreatment, 6 months, and 12 months, and consisted of ratings by blinded independent assessors and patient self-report. The results showed that the DBT-oriented treatment produced significantly greater gains than CCT on measures of suicide and self-harm, impulsiveness, anger, depression, global mental health, and on hospitalization days, although not on anxiety. Although three of the 12 patients (25%) in the DBT condition and 6 of 12 in the CCT condition (50%) dropped out of treatment, all patients participated at all assessments.

***Treatment integrity.*** Relevant to construct validity, it should be noted that a modified form of DBT was used, referred to as “DBT-oriented therapy”. There were two modifications to standard DBT: (1) there were no separate DBT skills training groups; instead training of skills was provided during the individual therapy sessions; and (2) “psychodynamic techniques” were incorporated to conceptualize the patients’ ”behavioral, emotional and cognitive relationship schema” (p. 415). Turner (2000a, p. 415) reports that the therapists had 3 months theoretical and practical training in each form of treatment, and that two supervisors (“the investigator” and a “senior clinical psychologist”) monitored adherence to the treatment protocols; the therapists presented audiotapes of their previous sessions, and the supervisors coached them to return to the protocol when therapists deviated from the assigned treatment plan. No data are reported on adherence, competence, or differentiation, thereby producing a TTI of .25 (see Table 1).

***Therapist experience***. The same four therapists carried out both treatments. Turner (2000a, p. 414) describes the four therapists as “regular mental health staff in a community mental health clinic” with an average of 22 years of experience. The therapists are described as having “theoretical backgrounds in family-systems, client-oriented, and psychodynamic therapy” (p. 415), which suggests the possibility that they may have had more experience with CCT than DBT; no data, however, are reported on this. Because the same therapists carried out both treatments, therapist experience was coded as “no evidence of a difference”.

***Dosage****.* To keep the treatment conditions equal with regard to clinical contact hours, Turner (2000a) decided not to run a separate DBT skills training group, but to provide skills during the course of individual therapy. In addition, six group sessions were provided to patients in both treatment conditions. In terms of design, therefore, there were no differences in dosage. Although more patients in DBT than in CCT completed 12 months of treatment (75% vs. 50%), Turner reports that there were “no significant differences between groups regarding the average number of treatment sessions during either the first or last 6-month periods” (p. 415). Accordingly, dosage was coded as “no evidence of a difference”.

***Supervision***. The supervision arrangements were similar, as two supervisors met with the therapists weekly in two separate group supervision meetings. This factor was therefore coded as “no evidence of a difference”.

***BPD-specific rationale***. Both treatments were manualized, DBT according to Linehan’s (1993) manual (with the two above-mentioned modifications) and CCT according to Carkhuff et al.´s (1976) protocol. Only the DBT manual, however, is based on a BPD-specific theoretical rationale. This factor was therefore coded as higher for DBT.

***Empathy, validation and support***. Support, empathy and validation are central to the DBT model (Linehan, 1993). The same, however, is true of CCT; as stated by Turner (2000a), the Carkhuff manual “provide directions for increasing the therapeutic relationship's empathic and supportive elements” (p. 416) and “accentuated the therapist's role as a supporter and advocate” (p. 416). In terms of empirical data, when the patients were asked to complete Luborsky’s (1984) l1-item Helping Relationship Questionnaire (HRQ) at the 6-month evaluation, there was no significant difference between treatments on the HRQ, *p* = .31. To summarize, this factor was therefore coded as “no evidence of a difference”.

***Medication***. At the beginning of the study, nineteen of the 24 patients were on medication, eleven in the CCT condition and 8 in the DBT condition; this difference, however, was not statistically significant, *p* = .132. At the 12-month evaluation, 4 DBT patients and 10 CCT patients reported they were receiving pharmacotherapy; this difference was significant, *p* = .01. Unfortunately, however, no data are reported on the use of medication *during* treatment. This factor was therefore coded as “no data reported”.

***Allegiance***. In a previous article, Turner has written in support of DBT (Turner, Barnett & Korslund, 1999). This factor was therefore coded as an allegiance in favor of DBT.

***Summary***. As described above, four of the seven alternative treatment contrasts (therapist experience, dosage, supervision, and empathy/validation/support) showed no evidence of a difference, thereby producing an ATCI of .57. Apart from the experimental contrast (i.e., DBT vs. CCT), this leaves at least two alternative contrasts as possibly contributing to the superior effects of DBT: (1) the use of a clear BPD-specific rationale, and (2) a researchers’ allegiance in favor of DBT.

**Study 2. DBT vs. Comprehensive Validation Therapy (Linehan et al., 2002).**

The participants in this study were 23 heroin-dependent women with BPD. The study had a component analysis design, comparing one year of DBT with one year of Comprehensive Validation Therapy (CVT), a manualized form of treatment which contained the acceptance/ validation strategies from DBT without its behavioral change components. One stated reason to conduct this study was that “[e]fforts to get behavior therapy to work with BPD in our clinic were not effective until comprehensive validation was added to the treatment, suggesting that validation may be the key treatment factor” (Linehan et al., 2002, p. 16). To balance the use of skills training groups in DBT, the CVT condition was combined with a 12 step program in accordance with Narcotics Anonymous policy. Eleven patients were randomized into DBT and 12 into the CVT+12S condition.

***Outcome***. Both treatments were associated with equivalent reductions in psychopathology, and there were no differences in parasuicidal behavior during the treatment year (p. 22). Urinalyses showed a similar course of declining opiate use in both treatment conditions up until the 8-month point, at which time the CVT+12S subjects showed a course of increasing opiate use while the DBT subjects maintained treatment gains; the 4-month follow-up, however, “showed no significant between-condition difference, with a low percentage of positive urinalyses in both conditions (DBT =/27%; CVT+12S = 33%)” (p. 20).

***Treatment integrity****.* Both treatments were manualized. The CVT+12S is described as a manualized form of treatment which includes “all of the DBT acceptance-based strategies, including validation, reciprocal communication (e.g., warmth, self-disclosure, responsiveness, and genuineness), and environmental intervention when requested (e.g., case management)” (p. 16), without its behavioral components. In fact, the CVT treatment “proscribed use of cognitive-behavioral change techniques or any overt suggestion of new behaviors or advice about what to do” (p. 16). The authors report that “[t]o promote adherence to treatment manuals, therapists in each condition met weekly with supervisors to discuss case material and review session videotapes” (p. 17). No measures of treatment integrity were reported, however, resulting in a TTI of .25 (see Table 1).

***Therapist experience***. Two doctoral-level and one masters level behavior therapist delivered DBT, and two master’s level therapists with chemical dependency certification and 12-step experience delivered the CVT+12S. All therapists had a minimum of 8 months training, but no comparison of therapist experience is reported. This factor was therefore coded as “no data reported”.

***Dosage****.* Significantly more patients dropped out of the DBT treatment (36%) than from the CVT treatment (0%). The authors, however, report “no significant difference in the mean number of individual sessions received (DBT: M =/33.2 ±/20.4; CVT+12S: M =/33.00 ±/S.D. =9.6) across the treatment year” (p. 19). On the other hand, “participants in the DBT condition attended a significantly greater number of skills group sessions (M =/26.6 ±/15.9) as compared with attendance of the CVT+12S participants in the 12-Step group meetings (M =/10.8 ±/12.8; *t*[21] =/2.62, *p <*/0.05). Likewise, the DBT patients attended a significantly greater number of individual coaching sessions (M =/17.6 ±/9.9) when compared to the number of 12-step sponsor sessions attended by those in the CVT+12S condition (M =/6.7 ±/2.5; *t*[21] =2.30, *p* </0.05).” (p. 19). The dosage was therefore coded as higher in DBT.

***Supervision***. Therapists in both conditions met weekly with supervisors to discuss case material and review session videotapes. This factor was therefore coded as “no evidence of a difference”.

***BPD-specific rationale***. Both DBT and CVT+1S are described as manualized forms of treatment. The CVT+12S treatment was based on a theoretical rationale which stated that both borderline personality disorder and drug abuse are “inevitable consequences of inability to self-validate” (p. 16). Thus, both treatments had a BPD-specific theoretical rationale. This factor is coded as “no evidence of a difference”.

***Empathy, validation and support***. The study was designed to keep this factor constant across the treatment conditions, but no patient ratings are reported. This factor was coded as “no evidence of a difference”.

***Medication***. In addition to psychotherapy, all participants received concurrent opiate agonist therapy three times per week, with “no between-condition differences in dosages” (p. 17). Common to both treatments were also optional psychotropic medication (p. 16), but no comparison is reported in usage between the treatments. Because the conditions did not differ on opiate agonist therapy, this factor was coded as “no evidence of a difference”.

***Allegiance***. Applying Munder’s (2011) indicators shows that, although Linehan developed both treatments, there was a researchers’ allegiance in favour of DBT, in the sense that Linehan primarily advocates DBT, and favors an etiological model consistent with DBT.

***Summary***. This study apparently managed to eliminate four of seven treatment contrasts (BPD-specific rationale, supervision, empathy/validation/support, and medication), rendering it an ATCI of .57. The dosage and allegiance factors were both in favor of DBT.

**Study 3. SFT vs. TFP (Giesen-Bloo et al., 2006; Spinhoven et a., 2007).**

In this study, 88 patients were randomized to three years of either schema-focused therapy (SFT; Young et al., 2003) or transference-focused psychotherapy (TFP; Clarkin et al., 1999) with 50-minute sessions twice a week. Randomization was stratified across 4 community mental health centers in the Netherlands, and was performed by a study independent person. The patients were assessed every 3 months for 3 years by independent research assistants.

***Outcome***. Assessments were made pre-treatment and every 3 months for 3 years by independent research assistants. Outcome data were available for 44 SFT patients, and 42 TFP patients. After 3 years of treatment, the SFT patients showed significantly larger improvements on most measures, including overall psychopathology, personality, parasuicidality, and quality of life.

***Treatment integrity****.* Treatment integrity was monitored by means of supervision, and assessed by other trained therapists for each orientation, who rated the adherence and competence on specifically developed scales with an identical cutoff score of at least 60. The median global competence rating of the therapists was 73 in the SFT condition, and 65 in the TFP condition. The authors also report that a psychologist, who was blind to allocation, listened to one randomly selected taped session from each patient, and was able to correctly classify 85 of 86 tapes (Giesen-Bloo et al., 2006, p. 651). Although competence was rated as satisfactory for both treatments, the higher competence ratings for SFT than for TFP represent a possible threat to treatment-construct validity. In all, this clearly indicates that the treatments differed, although the equality of competence of the therapists may be questioned (TTI = 0.75).

***Therapist experience***. At an average the SFT therapists had worked 9.95 (SD 4.98) years in their orientation with BPD patients; the corresponding figure for the TFP therapists were 11.73 (SD 6.28) years). This difference was not significant (*p* =.39), and the therapist factor was therefore coded as “no evidence of a difference”.

***Dosage****.* Eleven of the 44 SFT patients (25%) and 21 of the 42 TFP patients (50%) dropped out of treatment, which suggests that the TFP patients may have received less treatment. Giesen-Bloo et al. (2006), however, do not report any overall comparison of dosage. Instead they report the number of sessions separately for completers and dropouts. Although the dropout patients in the SFT condition had significantly more sessions than the dropout patients in the TFP condition (median: 98 vs. 34; *p* <.001), the opposite was the case for the completer patients: The completers in the SFT condition had significantly fewer therapy sessions than the completers in the TFP condition (median: 189.5 vs. 231.0; *p* =.002). Because no overall comparison of dosage is made between the treatments, the dosage factor is coded as “no data reported”.

***Supervision***. Throughout the study, there was weekly local supervision in groups with 4 to 5 SFT or TFP therapists, and also central supervision by two of the originators of the treatment, Jeffrey Young for SFT and Frank Yeomans for TFP, 1-day every 4 months, and 2-days every 9 months. This factor was therefore coded as no evidence of a difference.

***BPD-specific rationale***. Both treatments are manualized, with treatment protocols that address treatment frame, different phases, and the use of strategies and techniques. Both treatments are also based on a specific theoretical model about the development and treatment of BPD. This factor was therefore coded as no evidence of a difference.

***Empathy, validation and support***. As described by Spinhoven et al. (2007), the SFT therapist develops a supportive relationship which aims, among other things, to provide “limited reparenting” to meet some of the patient’s unmet emotional needs, whereas in TFP “an active supportive relationship with the patient is considered contraproductive”, as supportive interventions “are considered to interfere with the development of the negative transference or – in a less harsh scenario – to blur the negative transference, creating an as-if world and making the negative transference less amenable to therapeutic interventions” (p. 104-105). This indicates that the SFT model prescribes a more supportive and validating therapeutic style than the TFP model. In this context, it is also relevant to note that the patients after 3 months, after 15 months, and after 33 months completed a revised version of the Working Alliance Inventory (WAI-P; Horvath & Greenberg, 1989), measuring the contribution of the therapist to the alliance as perceived by the patient, and the SFT patients scored higher on the WAI-P than the TFP patients. Altogether, this indicates that the SFT therapists used more support and validation than the TFP therapists.

***Medication***. Psychiatrists evaluated the patients’ medications at the start of treatment and regularly during treatment, “prescribing according to good clinical practice, similar to American Psychiatric Association guidelines” (Giesen-Bloo et al., 2006, p. 651). At baseline, 77.3% of the SFT patients were on psychotropic medication vs. 71.4% of the TFP patients, which was not statistically significant. The use of medication tended to decrease during treatment, but “[t]he treatment group X medication interaction was not significant” (p. 654). Medication was therefore coded as “no evidence of a difference”.

***Allegiance***. Applying Munder et al.’s (2011) criteria, the authors show an allegiance for the SFT model, as one of the authors (Arntz) has written two books in favor of schema therapy (Arntz & Jacob, 2013; Arntz, van Genderen, Drost, Sendt, & Baumgarten-Kustner, 2009).

***Summary***. As described above, data on four of seven alternative contrasts (therapist experience, a BPD-specific rationale, supervision and medication) suggest that these did not differ, resulting in an ATCI of .57. Remaining as potential contributing factors to the superior outcome of SFT were, apart from the experimental contrast (SFT vs. TFP), differences in therapist competence, a larger use of support and validation in SFT, and a researchers’ allegiance in favor of SFT.

**Study 4. DBT versus community treatment by experts (Linehan et al., 2006; Bedics, Atkins, Comtois, & Linehan, 2012)**.

The participants were 101 women aged 18-45 years, with a diagnosis of BPD and the presence of suicidal attempts or self-injurious behavior. Fifty-two of the participants were randomized to DBT, and 49 to “community treatment by experts” (CTBE). The expert therapists were nominated by community mental health leaders (e.g., heads of inpatient psychiatric units and clinical directors of mental health agencies) as being especially skillful in treating difficult clients. In addition, to be included as therapists in this study, they had to be psychodynamically or eclectically oriented; no cognitive behavior therapists were selected for the expert condition. The expert therapists were simply asked to provide the type and dose of therapy that they believed was most suitable for the patient, with a minimum of one scheduled individual session per week. Ancillary treatment could also be prescribed as needed.

***Outcome***. Assessments were made by blinded independent clinical assessors at pre-treatment and at 4-month intervals during the treatment and follow-up periods. DBT was associated with better outcomes than the CTBE therapists on most reported measures, including suicide attempts, hospitalization for suicide ideation, and lower medical risk across all suicide attempts and self-injurious acts combined. Also, using the Intrex Short Form of the Structural Analysis of Social Behavior (SASB; Benjamin, 1983), Bedics et al. (2012) found that the DBT participants developed a more positive self-image (a more affiliative introject) than those treated by the CTBE therapists, including significantly greater self-affirmation, self-love, self-protection, and less of self-attack.

***Treatment integrity****.* Treatment adherence was monitored by supervision. Also, the DBT therapists’ adherence was found to be generally good, based on an assessment of 51 therapist-client dyads across 571 sessions. Although the characteristics of the therapists treatment in the CTBE were controlled via the selection procedure (i.e., no CBT therapists were included), the treatment in this condition was uncontrolled by the research team. That is, no data on treatment differentiation were reported. This resulted in a TTI of .00 (see Table 1).

***Therapist experience***. Fourteen of the 25 CTBE therapists (56%) had more than 10 years clinical experience after terminal degree, as compared with 4 of the 16 DBT therapists (25%). Linehan et al. (2006) report that the CTBE therapists had more clinical experience than the DBT therapists (*p* = .06); this factor was therefore coded as evidence of a difference between the treatments.

***Dosage****.* DBT was more effective in keeping subjects in treatment, as reflected in a 25% dropout rate vs. 59% in the CTBE condition. The patients in the DBT condition received significantly more individual therapy sessions from their study therapists than those in the CTBE condition (42.5 vs. 33.0), and also differed from the CTBE patients by having weekly group sessions. Although the authors report that there was no significant difference in total hours of treatment when non–study-provided treatment hours (group, individual, case management, day treatment, and inpatient treatment) were included, this comparison seems to include other forms of treatment than psychotherapy. The therapy dosage was therefore coded as higher in DBT.

***Supervision***. Group clinical consultations were a regular part of the supervision of the DBT therapists. Although the CTBE therapists were offered supervision at the prestigious Seattle Psychoanalytic Society and Institute, they were not required to attend any weekly clinical supervision group. Significantly more clients in the DBT condition than in the CTBE condition (100% vs. 51.7%, *p* < .001) had therapists who attended group clinical consultations. This factor was therefore coded as higher in DBT.

***BPD-specific rationale***. DBT followed Linehan’s (1993) manual. Nothing is known, however, about the extent to which therapists in the control condition used a BPD-specific theoretical rationale; the only information provided is that the CTBE therapists “were asked to provide the type and dose of therapy that they believed was most suited to the patient” (LInehan et al., 2006, p. 359). This factor was therefore coded as “no data reported”.

***Empathy, validation and support***. The patients rated the therapist’s behavior on the Intrex Short Form of the Structural Analysis of Social Behavior (SASB; Benjamin, 1983) at 4 months, 8 months and 12 months during treatment. Using these data, Bedics et al. (2012) found that DBT patients reported their therapists “as increasingly more affirming, protecting, and controlling during treatment compared to CTBE therapists” (p. 71). Because of the significant difference on “affirming” and “protecting” this factor was rated as higher in DBT.

***Medication***. As reported by Linehan et al. (2006), there were no differences between the treatment conditions in the use or the types of psychotropic medications at pretreatment, but during the treatment year the use of psychotropic medications decreased significantly more in the DBT group than in the expert-treated group. The use of medication was therefore coded as differing in the opposite direction to that of treatment efficacy.

***Allegiance***. Applying Munder et al.’s (2011) indicators, there was a clear researchers’ allegiance in favor of DBT: the first author (Linehan) developed DBT, advocates DBT, and has contributed to an etiological model which is consistent with DBT.

***Summary***. All analyses of treatment contrasts showed evidence of differences between the treatments, producing an ATCI of .00. Two factors, however, differed in the opposite direction to treatment outcome (therapist experience and medication), thereby making these factors unlikely to be causally involved in the outcome. Remaining as potential causal factors, apart from the experimental contrast (DBT vs. CTBE), were dosage, supervision arrangements, borderline-specific rationale (which, however, could not be supported by the data), degree of empathy/support/validation, and a clear researchers’ allegiance in favor of DBT.

**Study 5. TFP vs. DBT vs. supportive psychodynamic treatment (Clarkin et al., 2007; Levy et al., 2006)**.

In this study, 90 BPD patients were randomized to one year of TFP, DBT, or supportive psychodynamic treatment (SPT). SPT was psychodynamic, but differed from TFP in proscribing transference interpretations (i.e., the intended active ingredient in TFP). Although the therapists provided treatment in their private offices in the community rather than in a university or hospital setting, the standard DBT and TFP format were preserved. That is, DBT consisted of weekly individual therapy and group skills training, as well as available telephone consultation, and TFP consisted of two individual weekly sessions. SPT consisted of only one weekly session, but this could be supplemented with additional sessions as needed. Each of the three psychotherapies was administered and supervised by a treatment condition leader, who was an acknowledged expert on this treatment (Frank E. Yeomans in TFP, Barbara Stanley in DBT, and Ann Appelbaum in SPT).

***Outcome***. There were no significant differences between the treatments on any measure of borderline psychopathology, and Clarkin et al. (2007) conclude that the treatments “are generally equivalent with respect to broad positive change in borderline personality disorder” (p. 927). However, there were some tendencies. First, there was a non-significant tendency (*p* < .07, one-tailed) for DBT and TFP to be more effective than SPT on the measure of suicidality. Second, individual growth curve analysis showed that TFP predicted significant (*p* < .05) improvement in 10 of 12 variables, whereas DBT predicted change in only five of these 12 variables, and supportive treatment on six of these 12 variables. Of particular interest here is that TFP predicted change in anger and verbal assault even with Bonferroni correction (*p* < .001), whereas the other treatments did not. Further, in a separate analysis of ratings from transcribed Adult Attachment Interviews, Levy et al. (2006) found that TFP patients (but not those in the other two conditions) improved significantly on two measures of mentalization (narrative coherence and reflective functioning), and showed a significant increase in the percentage of individuals classified as secure in their attachment pattern.

***Treatment integrity***. All therapists attended weekly group supervisions in which the treatment condition leaders were able to observe videotaped sessions, and provide feedback “if a therapist fell below an acceptable level of either adherence to the manual or competence” (Levy et al., 2006, p. 1032). Further, “when a therapist’s ratings were consistently low for adherence, then ratings were made more frequently (approximately every four sessions) for the succeeding 3-month interval, and supervision focused on the difficulties identified by raters. Additional individual supervision was provided when either adherence and/or competence fell below acceptable levels. When a therapist fell below acceptable levels, no new cases were assigned to them.” (Levy et al., 2006, p. 1032). No data on treatment integrity, however, are reported, resulting in a TTI of .25 (see Table 1).

***Therapist experience***. There is little information about the therapists’ competence or experience, other than that they “were selected by the treatment condition leaders based on prior demonstration of competence in their respective modality” (Clarkin et al., 2007, p. 923) and that all therapists had “at least 2 years of prior experience treating patients with borderline personality disorder” (p. 923). This factor was coded as “no data reported”.

***Dosage****.* There are no data on the number of actual sessions that the patients had in each treatment condition, nor is there any explicit information about how many patients dropped out of treatment. Dosage is therefore coded as “no data reported”.

***Supervision***. All therapists attended weekly group supervisions. This factor is therefore coded as “no evidence of a difference”.

***BPD-specific rationale***. Both DBT (Linehan, 1993) and TFP (Clarkin et al., 1999) are manualized treatments based on a clear BPD-specific theoretical rationale. SPT is described as “a manualized psychoanalytically oriented treatment for borderline patients” (Levy et al., p. 1031), developed by the treatment condition leader (Appelbaum, 2005), adapted from Rockland’s (1992) psychodynamic approach to supportive therapy for borderline patients. This factor is therefore coded as “no evidence of a difference”.

***Empathy, validation and support***. No data are reported. To judge from the manuals, however, validation and support are more strongly emphasized in DBT and in SPT than in TFP. This factor was therefore coded as higher in DBT and in SPT than in TFP.

***Medication***. As reported by Clarkin et al. (2007), medication “was decided on an individual basis so that the use or nonuse of medication and medication type and amount were not standardized” (p. 923). At treatment onset, 70% of the DBT patients, 65% of the SPT patients, and 52% of the TFP patients were on medication, and the “percentage of patients receiving medication remained relatively constant throughout the 1-year treatment period” (p. 924). Test by chi-square shows that 70% compared to 52% in two samples of 30 participants is not statistically significant; the medication factor was therefore coded as “no evidence of a difference”.

***Allegiance***. There is a clear researchers’ allegiance, as the main originators of the TFP model, Kernberg and Clarkin, are co-authors of both papers.

***Summary***. To summarize, three of the seven alternative treatment contrasts (BPD-specific rationale, supervision, and medication) were coded as “no evidence of a difference”, which resulted in an ATCI of .43. Two other factors (empathy/support/validation and allegiance) were coded as different, although in opposite directions: more focus on empathy, support and validation in DBT and SPT, and an allegiance in favor of TFP.

This trial presented us with some problems in terms of coding it as “equal efficacy” or “differerential efficacy”. In terms of BPD symptom change, there were no significant differences between these three treatments. However, TFP and DBT showed a non-significant tendency (*p* < .07, one-tailed) to be more effective than SPT on the measure of suicidality, and individual growth curve analysis showed that TFP predicted significant improvement in more variables than DBT and SPT (10 vs. 5 vs. 6, respectively). Further, a separate analysis of ratings from transcribed Adult Attachment Interviews showed more evidence of change among TFP patients than among DBT and SPT patients on measures of mentalization and attachment. The trial *might* therefore also have been categorized as a differential efficacy comparison.

**Study 6. MBT vs. Structural Clinical Management** **(Bateman & Fonagy, 2009).**

In this study, 134 outpatients with BPD were randomized either to Mentalization-Based Treatment (MBT, N =71) or a control condition referred to as Structural Clinical Management (SCM, N =63) which was created for this particular study. All patients were offered 18 months of therapy, and about 75% of the patients met the criteria for completion (defined as at least 70 sessions attended over the first year).

***Outcome***. The MBT patients showed a better outcome than the SCM patients on a number of measures, including depression, social adjustment, suicide at­tempts and severe self-injuries.

***Treatment integrity****.* Treatment integrity was measured with separate adherence rating scales; average ratings yielded 85% adherence to MBT and 96% adherence to the SCM manual (Bateman & Fonagy, 2009, supplementary data, p. 1). No data are reported, however, on mentalization-focused interventions in SCM, or on supportive and problem-oriented interventions in MBT treatment; the data reported, therefore, do not allow any conclusion on the extent to which the treatments differed. The TTI was rated as .50 (see Table 1).

***Therapist experience***. The therapists did not differ in their years of psychiatric experience: 6.15 (SD 1.6) years for the MBT therapists, and 6.8 (SD 2.3) years for the SCM therapists (Bateman & Fonagy, 2009, supplementary data, p. 3). This factor was therefore coded as “no evidence of a difference”.

***Dosage****.* Bateman and Fonagy (2009) report that “[t]he mean number of scheduled clinical meetings attended was 92 (SD=38) for MBT and 84 (SD=40) for SCM, a nonsignificant difference” (p. 1359). Dosage was coded as “no evidence of a difference”.

***Supervision***. Supervision arrangements were similar (Bateman & Fonagy, 2009, supplementary data, p. 3). This factor was coded as “no evidence of a difference”.

***BPD-specific rationale***. Both treatments were manualized, and conducted within a structured frame­work following NICE Guidelines, which included crisis con­tact and crisis plans, and pharmacotherapy. There is nothing to suggest, however, that SCM was based on a theory about the etiology and treatment of BPD (Bateman, Fonagy, Bolton, & Karas, 2009). As stated by Bateman and Fonagy (2009), the superiority of MBT over CSCM may have been due to this difference in theoretical consistency, because “therapists are supported by a primary theoretical model onto which they consistently map clinical and interac­tional data. Without this, therapy may become incoher­ent and therapists responsive rather than proactive” (p. 1362-1363). This factor was therefore coded as higher in MBT

***Empathy, validation and support.*** The importance of empathy, validation and support is emphasized in both manuals. This factor was coded as “no evidence of a difference”.

***Medication***. Medication was as­sessed at baseline and at 6-month intervals until the end of treat­ment. At baseline, 77.5% of the MBT patients and 68.3% of the SCM patients were on medication, and the figures were similar at 6 months; after 12 months of treatment they had changed to 49.3% versus 61.9%, and at 18 months of treatment to 29.6% versus 57.2%. The reduction was significantly greater for the MBT group. That is, the use of medication differed in the opposite direction to that of treatment efficacy.

***Allegiance***. Because the authors have developed MBT, and the SCM model was created as a comparison condition for the present study, there is a researchers’ allegiance in favor of MBT.

***Summary***. This study showed no evidence of a difference on four of the seven alternative contrasts (therapist experience, dosage, supervision, and empathy/validation/support), rendering an ATCI of .57. Remaining as possible contributing factors to the superior outcome of MBT, apart from the experimental contrast (MBT vs. SCM), were two alternative contrasts in favor of MBT (a BPD-specific rationale, and a researchers’ allegiance in favor of MBT).

**Study 7. DBT vs. General Psychiatric Management (McMain et al., 2009; 2012)**.

In this study, 180 patients diagnosed with BPD were randomly assigned to receive 1 year of DBT or general psychiatric management (GPM). The latter is described as “a well-specified treatment delivered by psychiatrists with expertise in the treatment of borderline personality disorder” (McMain et al., 2009; p. 1365), and “consisted of case manage­ment, dynamically informed psychotherapy, and symptom-targeted medication management” (p. 1367).

***Outcome***. Of the 180 participants, 111 (62%) completed 1 year of treatment. Thirty-five (39%) patients in the DBT condition and 34 (38%) in the GPM condition ended treat­ment prematurely. Despite the statistical power due to the relatively large sample, no significant differ­ences were found between the groups on any outcome measure. Further, as stated by McMain et al. (2009), “the effect sizes between groups were negligible, without any indication of trends toward differences” (p. 1373). As reported in McMain et al.’s (2012) 2-year follow-up, both treatment groups also showed equal effects on the majority of outcome measures 2 years after discharge.

***Treatment integrity****.* DBT was implemented in its standard form. The GPM condition included psychodynamic therapy according to Gunderson and Link’s (2008) approach, with an emphasis on relational aspects, and the only “primary strategy” which is mentioned by McMain et al. (2009) as exclusive for GPM is “active attention to negative transference” (p. 1367, Figure 2). Modality-specific adherence scales were used to evaluate treat­ment integrity, and adherence was supported for both conditions; adherence ratings for DBT were reported as “comparable” (p. 1372) to those reported by Linehan et al. (2006). Importantly, the mean adher­ence scores for essential interventions in the GPM treatment condition were significantly greater than the mean adherence score for proscribed DBT items across all time points (*p*<0.001). To check further for the contamination of DBT techniques into GPM, “a random sample of general psychiatric management sessions (N=10) was rated on the dialecti­cal behavior therapy adherence scale. Overall scores fell below adherence (<4.0), lending support to the idea that general psychiatric management was not a version of dia­lectical behavior therapy” (p. 1373). The data therefore indicate good treatment integrity (TTI = .75).

***Therapist experience***. There was no difference in mean years of clinical experience between the DBT therapists (15.0 [SD=9.58]) and the GPM therapists (14.2 [SD=9.97]).

***Dosage****.* The DBT patients attended more weeks of treatment (i.e., weeks with at least one session) than the GPM patients (36 weeks [SD=17.57] compared with 27 weeks [SD=14.84]; *p*<0.001). Although there was no significant difference in the mean number of individual sessions (32 [SD=15.97]) versus 31 [SD=27.05]), the DBT patients in addition attended a mean of 26 (SD=14.98) group therapy sessions (whereas no group sessions were used in GPM). The dosage factor was therefore coded as higher in DBT.

***Supervision***. The DBT therapists had 2 hours regu­lar weekly supervision, supplemented by consultation from interna­tional experts. The GPM therapists similarly attended 90 minutes weekly group supervision, supplemented by consultation from interna­tional experts. This factor was coded as “no evidence of a difference”.

***BPD-specific rationale***. Both treatments were manualized and used psychoeducation as a primary strategy (McMain et al., 2009, p. 1367). The DBT condition followed Linehan’s (1993) model, and GPM followed an approach from Gunderson and Links (2008), based on the assumption that disturbed attachment relationships related to emotion dysregulation is a primary deficit in BPD. Both treatments included “protocols designed to de-escalate suicide crises” (McMain et al., 2012, p. 659). This factor was coded as “no evidence of a difference”.

***Empathy, validation and support***. Both treatments are described as using empathy and validation as a primary strategy (McMain et al., 2009, Figure 2). This factor was therefore coded as “no evidence of a difference”.

***Medication***. As reported by McMain et al., (2009), “[t]o maximize external validity, there were no restrictions on ancillary pharmacotherapy” (p. 1367). In the DBT condition, patients were encouraged to rely on “skills over pills” where appropriate (e.g., anxiolytics), but psychopharmacologic intervention was uncontrolled. In the GPM condition, patients were “encouraged to use medications concurrently. Two medication algorithms, one related to mood lability and one related to impulsive-aggressiveness, were prioritized as symptom targets. Medication intervention was delivered according to the predominant symptom pattern.” (p. 1367) Despite these different policies, there were no significant group differences in medication, neither during treatment (McMain et al., 2009, p. 1371) nor during follow-up (McMain et al., 2012, p. 3-4).

***Allegiance***. The authors of both studies include representatives of both DBT (Shelley McMain) and GPM (Paul Links). No evidence of allegiance was coded.

***Summary***. This study apparently managed to eliminate six of seven factors (therapist experience, BPD-specific rationale, supervision, empathy/support/ validation, medication, and researchers’ allegiance), resulting in an ATCI of .86.

**Study 8. TFP vs. experienced therapists (Doering et al., 2010).**

In this study, which was carried out at university clinics in Munich, Germany, and Vienna, Austria, 104 BPD patients were randomly assigned to 1 year of TFP or treatment by experienced community psychotherapists. The latter were a very heterogeneous group (19 patients were treated by psychoanalysts, 18 by behavior therapists, 4 by client-centered therapists, 4 by systemic psychotherapists, and 1 by a Gestalt therapist).

***Outcome***. The TFP condition produced significantly larger effects on most measures, including borderline symptomatology, psychosocial functioning, personality, psychiatric in-patient admissions, and attempted suicide.

***Treatment integrity****.* Four sessions of each therapy in the TFP condition were included in analyses of adherence and competence, and ratings are reported as “satisfactory”. No integrity checks were performed of therapies in the control condition (Doering et al., 2010, p. 391), however, resulting in a TTI of .00 (see Table 1).

***Therapist experience***. The TFP therapists had 9.4 (SD 7.9) years of experience after completion of their psychoanalytic/psychodynamic training, and the experienced community therapists had 8.9 (SD 9.8) years of experience after completion of their psychotherapy training (Doering et al., 2010, p. 391), which was not significantly different. This factor was therefore coded as “no evidence of a difference”.

***Dosage****.* The TFP patients received significantly more therapy sessions (48.5 vs. 18.6 sessions, p < .001). Dosage was therefore coded as higher in TFP.

***Supervision***. In the TFP condition, all sessions were video recorded for use in group supervision, either weekly for 2 hours or every fortnight for 4 hours. The experienced community psychotherapists “attended supervision according to their usual routine” (p. 391), and their sessions were not recorded. Because the TFP therapists received significantly more supervision than the experienced community therapists, 21.5 (SD = 8.5) sessions vs. 11.4 (SD = 12.3) sessions (*p* = .003), this factor was coded as higher for TFP.

***BPD-specific rationale***. TFP used a German translation of Clarkin et al.’s TFP manual, which is based on a clear BPD-specific theoretical rationale. No information is given about the structure of the wide variety of treatments that were practiced in the control condition although it is said that “none of the therapists had specific training in manualised borderline psychotherapy” (p. 391). This factor was coded as “no data reported”.

***Empathy, validation and support.*** No data are reported.

***Medication***. Medication was decided on an individual basis by the patients’ psychiatrists. There were no significant differences between the groups with regard to medication, neither at baseline nor during the 1-year treatment period. The medication was therefore coded as “no evidence of a difference”.

***Allegiance***. The first author (Doering) was appointed vice president of the International Society of Transference-Focused Psychotherapy when it was founded in 2011; there is thus a clear researcher’s allegiance in favor of TFP.

***Summary***. Two of the seven alternative contrasts (therapist experience and medication) were coded as “no evidence of a difference”, resulting in an ATCI of .29. Four other factors (dosage, a BPD-specific rationale, supervision, and allegiance) remain as possibly contributing to the superior outcome of TFP.

# References

American Psychiatric Association (2001). Practice guideline for the treatment of patients with borderline personality disorder. *American Journal of Psychiatry, 158* (October supplement).

Appelbaum, A. H. (2005). Supportive psychotherapy. In J. Oldham, A. Skodol, & D. Bender (Eds.), *The American Psychiatric Publishing* *textbook of personality disorders* (pp. 311–326). Washington, DC: American Psychiatric Publishing.

Arntz, A. & Jacob, G. (2013). ***Schema****therapy in practice: An introductory guide to the****schema****mode approach.* Wiley-Blackwell.

Arntz, A., van Genderen, H., Drost, J., Sendt, K., & Baumgarten-Kustner, S. (2009). ***Schema*** *therapy for borderline personality disorder.* Wiley-Blackwell.

Bateman, A., & Fonagy, P. (1999). Effectiveness of partial hospitalization in the treatment of borderline personality disorder: A randomized controlled trial. *American Journal of Psychiatry, 156,* 1563–1569.

Bateman, A. & Fonagy, P. (2004). *Psychotherapy of Borderline Personality Disorder: mentalization based treatment.* Oxford: Oxford University Press.

Bateman, A., & Fonagy, P. (2009). Randomized controlled trial of outpatient mentalization-based treatment versus structured clinical management. *American Journal of Psychiatry, 166*, 1355–1364.

Bateman, A., Fonagy, P., Bolton, R., & Karas, E. (2009). Structured clinical management for borderline personality disorder. Unpublished manuscript. Hallwick Unit, S:t Ann’s Hospital, London.

Bedics, J. D., Atkins, D. C., Comtois, K. A., & Linehan, M. M. (2012a). Treatment differences in the therapeutic relationship and introject during a 2-year randomized controlled trial of dialectical behavior therapy versus nonbehavioral psychotherapy experts for borderline personality disorder. *Journal of Consulting and Clinical Psychology, 80,* 66-77**.**

Benjamin, L. S. (1974). Structural analysis of social behavior. *Psychological* *Review, 81,* 392–425.

Carkhuff, R. R., Pierce, R., & Cannon, J. (1976). *The art of helping.* Amherst, MA: Human Resources Press.

Clarkin, J. F., Levy, K. N., Lenzenweger, M. F., & Kernberg, O. F. (2007). Evaluating three treatments for borderline personality disorder: A multiwave study. *American Journal of Psychiatry, 164,* 922–928.

Clarkin, J. F., Yeomans, F. E., & Kernberg, O. F. (2006). *Psychotherapy for borderline personality. Focusing on object relations.* American Psychiatric Publishing.

Doering, S., et al. (2010). Transference-focused psychotherapy v. treatment by community psychotherapists for borderline personality disorder: randomised controlled trial. *The British Journal of Psychiatry, 196,* 389–395.

Gunderson, J. G. & Links, P. S. (2008). *Borderline Personality Disorder: A Clinical Guide*. *Second edition.* Washington, DC.: American Psychiatric Press.

Levy, K. N., Meehan, K. B., Kelly, K. M., Reynoso, J. S., Weber, M., Clarkin, J. F., & Kernberg, O. F. (2006). Change in attachment patterns and reflective function in a randomized control trial of transference-focused psychotherapy for borderline personality disorder. *Journal of Consulting and* *Clinical Psychology, 74,* 1027–1040. doi:10.1037/0022-006X.74.6.1027

Linehan, M.M. (1993). *Cognitive.behavioral treatment of borderline personality disorder.* New York: Guilford Press.

Linehan, M.M. (1997). Validation and psychotherapy. In A. Bohart & L. Greenberg (Eds.), *Empathy reconsidered: New directions in psychotherapy* (pp. 353-392). Washington, D.C.: American Psychological Association.

Linehan, M. M., Armstrong, H. E., Suarez, A., Allmon, D., & Heard, H. L. (1991). Cognitive-behavioral treatment of chronically parasuicidal borderline patients. *Archives of General Psychiatry, 48,* 1060–1064.

Linehan, M. M., Comtois, K. A., Murray, A. M., Brown, M. Z., Gallop, R. J., Heard, H. L., . . . Lindenboim, N. (2006). Two-year randomized controlled trial and follow-up of dialectical behavior therapy vs. therapy by experts for suicidal behaviors and borderline personality disorder. *Archives of General Psychiatry, 63,* 757–766.

Linehan, M. M., Dimeff, L. A., Reynolds, S. K., Comtois, K. A., Shaw-Welch, S., Heagerty, P., & Kivlahan, D. R. (2002). Dialectical behavior therapy versus comprehensive validation therapy plus 12-step for the treatment of opioid dependent women meeting criteria for borderline personality disorder. *Drug and Alcohol Dependence, 67,* 13–26.

McMain, S. F., Links, P. S., Gnam, W. H., Guimond, T., Cardish, R. J., Kormann, L., & Streiner, D. L. (2009). A randomized trial of dialectical behavior therapy versus general psychiatric management for borderline personality disorder. *American Journal of Psychiatry, 166,* 1365–1374.

McMain, S. F., Guimond, T., Streiner, D. L., Cardish, R. J., & Links, P. S. (2012). Dialectical behavior therapy compared with general psychiatric management for borderline personality disorder: Clinical outcomes and functioning over a 2-year follow-up period. *American Journal of Psychiatry, 169,* 650-661.

Munder, T., Gerger, H., Trelle, S., & Barth, J. (2011). Testing the allegiance bias hypothesis: hypothesis: A meta-analysis, Psychotherapy Research, 21:6, 670-684. doi.org/10.1080/10503307.2011.602752

NICE (2009). Borderline Personality Disorder: treatment and management. <http://www.nice.org.uk/Guidance/CG78/NiceGuidance/pdf/English>

Rockland, L. H. (1992). *Supportive therapy for borderline patients: A* *psychodynamic approach.* New York: Guilford Press.

Spinhoven, P., Giesen-Bloo, J., van Dyck, R., Kooiman, K., & Arntz, A. (2007). The therapeutic alliance in schema-focused and transference-focused psychotherapy for borderline personality disorder. *Journal of Consulting and Clinical Psychology, 75,* 104-115.

Turner, R. M. (2000a). Naturalistic evaluation of dialectical behavior therapy-oriented treatment for borderline personality disorder. *Cognitive* *and Behavioral Practice, 7,* 413–419.

Turner, R. M. (2000b). Understanding dialectical behavior therapy. *Clinical Psychology: Science and Practice, 7,* 95-98.

Turner, R. M., Barnett, B. E., & Korslund, K. E. (1998). The application of dialectical behavior therapy to adolescent borderline clients. *In Session: Psychotherapy in Practice*, *4,* 45-66.

Young, J. E., Klosko, J., & Weishaar, M. E. (2003). *Schema therapy: A* *practitioner’s guide.* New York: Guilford Press.
